# “Exosomics”—A Review of Biophysics, Biology and Biochemistry of Exosomes With a Focus on Human Breast Milk

**DOI:** 10.3389/fgene.2018.00092

**Published:** 2018-03-27

**Authors:** Carolina de la Torre Gomez, Renee V. Goreham, Joan J. Bech Serra, Thomas Nann, Martin Kussmann

**Affiliations:** ^1^Proteomics Unit, Bellvitge Biomedical Research Institute, Barcelona, Spain; ^2^MacDiarmid Institute for Advanced Materials and Nanotechnology, Victoria University of Wellington, Wellington, New Zealand; ^3^Liggins Institute, University of Auckland, Auckland, New Zealand; ^4^National Science Challenge “High-Value Nutrition”, The University of Auckland, Auckland, New Zealand

**Keywords:** exosome, breast milk, maternal health, proteomics, micronutrient

## Abstract

Exosomes are biomolecular nanostructures released from cells. They carry specific biomolecular information and are mainly researched for their exquisite properties as a biomarker source and delivery system. We introduce exosomes in the context of other extracellular vesicles, describe their biophysical isolation and characterisation and discuss their biochemical profiling. Motivated by our interest in early-life nutrition and health, and corresponding studies enrolling lactating mothers and their infants, we zoom into exosomes derived from human breast milk. We argue that these should be more extensively studied at proteomic and micronutrient profiling level, because breast milk exosomes provide a more specific window into breast milk quality from an immunological (proteomics) and nutritional (micronutrient) perspective. Such enhanced breast milk exosome profiling would thereby complement and enrich the more classical whole breast milk analysis and is expected to deliver more functional insights than the rather descriptive analysis of human milk, or larger fractions thereof, such as milk fat globule membrane. We substantiate our arguments by a bioinformatic analysis of two published proteomic data sets of human breast milk exosomes.

## Introduction

### What are exosomes?

Most living cells release an array of **extracellular vesicles** (EVs), i.e., membrane liposomes which are 20–200 nm in size. The nomenclature of the different vesicle types depends on their cell of origin, as well as their function and size, and has generated confusion about the definition of exosomes. The EV terms used in this expanding area of research encompass **exosomes**, ectosomes, microvesicles, microparticles, prostasomes, tolerosomes (which induce immunological tolerance to dietary antigens), apoptotic bodies (released by apoptotic cells), and nanovesicles. Cells deliver microRNA (miRNA), messenger RNA (mRNA), proteins, and other biomolecules between intracellular organelles by **membrane vesicles**, which contain receptors to ensure traffic specificity. These membrane vesicles are actively secreted by most cells and exist in most body fluids, including blood, saliva, breast milk and sperm. There are three main types of such membrane vesicles: microparticles, microvesicles (100–1,000 nm), and exosomes (20–200 nm) (Sun et al., 2010; Lawson et al., 2016).

**Exosomes** are bilayer membrane vesicles released by almost every mammalian cell type for intercellular communication and are unique to the cell of origin. For example, exosomal release from cancer cells contributes to metastasis through such intercellular communication. Exosomes are therefore used in theranostic applications, because they exhibit biomarker profiles specific to the diseased cell they are derived from.

Prior to exosome discovery, it was known that mammalian cells transmit information between cells indirectly, yet without this transmission being fully understood. In 1983, two independent papers reported that, in blood, reticulocytes were observed to mature into erythrocytes and that transferrin receptors were released into the space *via* small vesicles of 50 nm in size (Harding, 1983; Pan and Johnstone, 1983). Four years later, Rose Johnstone coined the term “exosomes” (Johnstone et al., 1987; Johnstone, 2005). The Nobel prize was awarded in 2013 to Ames E. Rothman, Randy W. Schekman and Thomas C. Südhof “for their discoveries of a machinery regulating vesicle traffic, a major transport system in our cells” (The Nobel Prize in Physiology or Medicine, 2013). This ultimately revealed the mechanism of exosome transport and triggered the potential use of exosomes as promising biomarkers for disease diagnostics and treatment. Immunology-related interest in exosomes did not manifest until 1996, when Raposo et al. (Raposo, 1996) found that B lymphocytes secrete exosomes carrying membrane-bound molecules essential for the adaptive immune response. Another report showed that also dendritic cells secrete exosomes (Delcayre et al., 2005), which carry functional immune agents promoting anti-tumor responses in mice (Zitvogel et al., 1998). Together, these results formed the basis for the hypothesis of intercellular communication *via* exosomes (Exosome Explosion | The Scientist Magazine®)^1^.

The growing interest in EVs, and particularly exosomes, is not only reflected by the increasing numbers of related scientific publications over recent years, but also by the creation of scientific associations, portals and databases, such as:

- ISEV: the International Society for Extracellular Vesicles (https://www.isev.org/);
- EU ME-HaD: the European Network on Microvesicles and Exosomes in Health and Disease (http://www.mehad-cost.eu);
- Vesiclepedia (http://www.microvesicles.org/); and
- ExoCarta (http://www.exocarta.org), created in 2009 as an open-access resource for compiling proteins and RNAs identified in exosomes (referenced in ‘Biochemistry of Exosomes’).

### Exosome biogenesis

The nomenclature of **exosomes** is critical (Bobrie et al., 2011). Gould et al. define the term exosome in three different ways: first, in terms of their biogenesis; second, with regard to their physiological function within cells; and third, with an empirical definition based on isolation by differential ultracentrifugation at 70,000–100,000 × g (Gould and Raposo, 2013). For clarity in this review, we define exosomes by their biogenesis.

**Figure 1:**
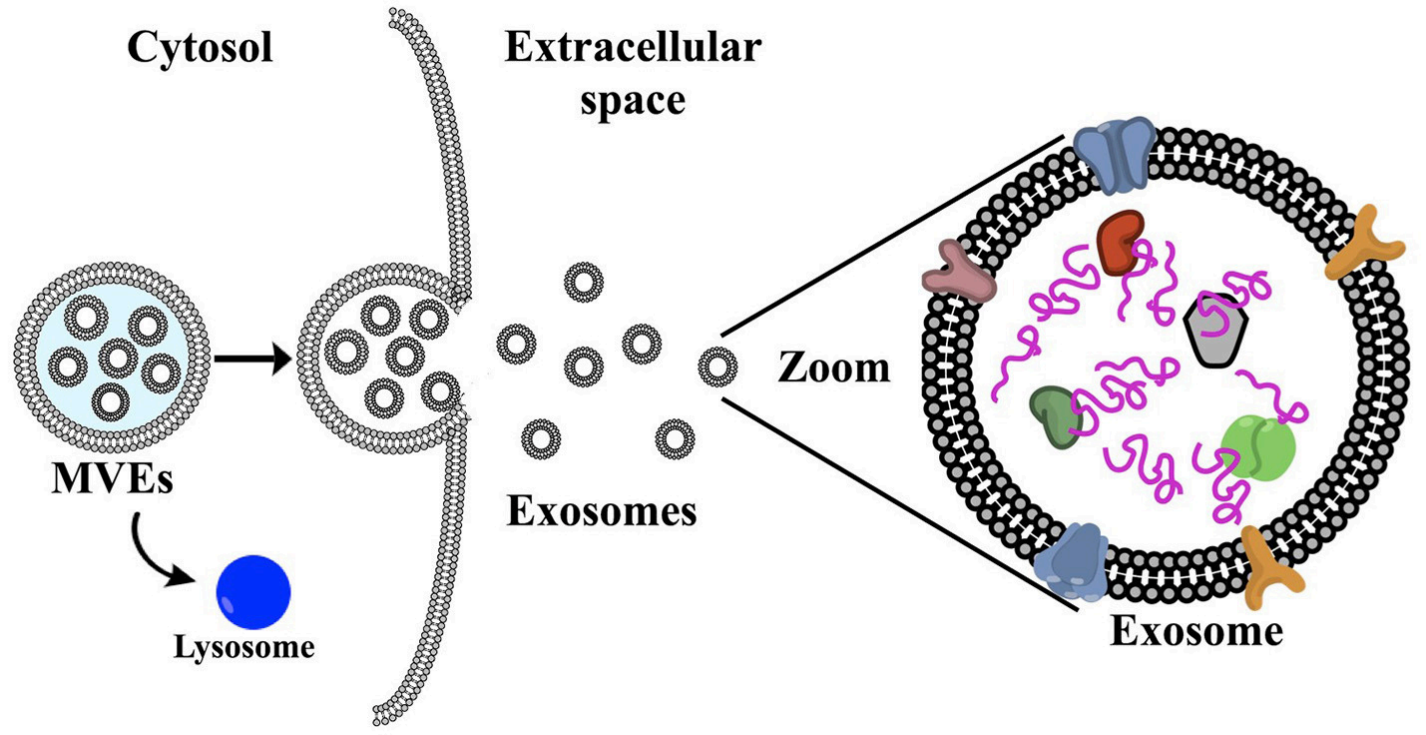
(a) Schematic representation of exosome biogenesis: first, multivesicular endosomes are formed (MVEs), which encompass the exosomes. The MVEs can either fuse with the plasma membrane, releasing the exosomes into the extracellular matrix (see zoomed schematic), or fuse with the lysosome for degradation. Microvesicles are formed *via* direct blebbing from the plasma membrane. Exosomes contain protein, DNA, RNA and surface membrane proteins, which are specific to the cell of origin and are not limited to cell surface proteins (Colombo et al., 2014).

Cells are experts at producing and exporting molecular products, for example, the transport of insulin into the bloodstream (Zheng et al., 2014). These molecules are submitted to a “cell packaging service,” i.e., the exosomes, which are first released intracellularly. Initially, **multivesicular endosomes** (MVEs) are formed, which encompass the exosomes (Figure 1). These intracellular exosomes are incorporated into the MVEs in the cytoplasm. Several mechanisms for exosome generation and release are described by Abels and Breakefield (2016). In essence, the MVEs fuse with the cell membrane releasing the exosomes to the extracellular matrix (Vlassov et al., 2012). Rab GTPases (peripheral membrane proteins) have been found to facilitate this fusion of MVEs with the plasma membrane, including RAB11 and RAB35 (Savina et al., 2003; Hsu et al., 2010), which in turn release exosomes enriched with flotillin and other cell-specific proteins. A second mechanism involves RAB27A and RAB27B, which promote the release of exosomes loaded with CD63 (a common target for specific capture of exosomes), TSG101 and ALIX (Abels and Breakefield, 2016). In contrast to exosomes, **microvesicles** are formed *via* direct blebbing from the plasma membrane, undergo a different mode of biogenesis, and include apoptic bodies. In summary, several complex pathways account for exosome generation from endosomes and the composition of the exosomes varies depending on the type and physiological state of the cell of origin.

### Exosome biophysics: isolation and characterization

Exosomes can be found in most human biological fluids, including blood, urine, saliva and breastmilk. Using exosomes as a biomarker source requires careful isolation and purification from surrounding biological fluids, which would otherwise interfere with the exosome analysis (see “Exosome Biochemistry”). A number of **exosome purification** methods have been developed with adaptation to the biological fluid from which the vesicles are derived. For example—and important to our focus on human breast milk—the storage conditions of milk have been shown to be an important factor for the final exosome concentration and integrity (Zonneveld et al., 2014). The most widely used isolation method is differential centrifugation, which selectively removes extracellular debris (Théry et al., 2006). However, this method typically generates rather low yields of exosomes, excess protein is still present, and the integrity of the exosomes is questionable. Another common method is solution sedimentation and low-speed centrifugation, inducing the precipitation of exosomes (van der Pol et al., 2014). Sucrose gradients are commonly employed to take advantage of the buoyant density in viscous fluids and to facilitate the isolation process (Oosthuyzen et al., 2013). Notably, all these aforementioned isolation methods are time-consuming and often expensive. Specifically for breast milk-derived exosomes, isolation and purification processes optimized for high yields at minimal time and cost are still lacking.

The biophysical characterisation of exosomes is typically limited to the determination of their **size and concentration**. For a benchmark in exosome isolation and characterisation of their size and concentration, the authors herewith refer to Lane et al. (2015). Most publications report measurements by dynamic light scattering (DLS). DLS determines the hydrodynamic radius and may not separate different populations (e.g., 100 and 200 nm), which can compromise the final value for mean size. The Nanosight technique is based on nanoparticle tracking analysis (NTA) for both size and concentration determination. This method measures the hydrodynamic radius, too, and comes therefore with the same limitations as DLS. A less recruited technology, especially for characterization of breast milk exosomes, is qNano, which uses tunable resistive pulse sensing (TRPS) and can measure single particles, thereby providing accurate particle size and concentration measurements.

Exosomes have a homogenous “cup-shaped” **morphology**, as determined by negative-staining electron microscopy (Théry et al., 2002; Simons and Raposo, 2009). For visualization of exosomes, transmission electron microscopy (TEM) can be deployed, but the low density of exosomes can limit the power of this technique. Moreover, if the samples are not highly pure, it can be difficult to distinguish between proteins, exosomes and other vesicles. Van der Pol et al. compared particle size distribution of urinary exosomes and microvesicles using TEM, flow cytometry, NTA and TRPS (van der Pol et al., 2014). They found that each method gives a different concentration and particle size distribution.

Important for our interest in biochemical characterisation of exosomes, Taylor et al. reported on exosome isolation specifically **optimized for proteomic analyses** and RNA profiling (Taylor et al., 2011): the exosomes isolated by different methods were analyzed in terms of quantity and quality of specific RNAs and marker proteins. ExoQuick™ precipitation of circulating exosomes produced RNA and protein with higher purity and quantity than chromatography, ultracentrifugation, and DynaBeads. While this precipitation method does not provide specificity of the originating cell, the high quantity and quality of exosomal proteins and RNA improve both sensitivity and accuracy of subsequent biomolecular characterisation, such as miRNA profiling and mass spectrometric proteomics.

In summary, exosomes are complex and delicate systems requiring optimized isolation and characterisation adapted to each fluid type of origin (e.g., breast milk, urine, blood). Their biophysical characterisation should deploy complementary instrumentation and methods rather than relying on a single measurement.

### Exosomes in health and disease

Since their discovery 30 years ago, exosomes have been found to play a vital role in many biological processes, including: intercellular communication; immune function; development and differentiation of stem cells; neuronal function; cell signaling; tissue regeneration; and viral replication (Rashed et al., 2017). Exosomes have been isolated from a variety of cell types *in vitro*. They have the ability to transfer molecular cargo and to be selectively taken up by specific cells, thereby reprogramming the target cell and, possibly, inducing disease. On the other hand, they can also provide new avenues for treatment and diagnosis.

The urgent need for clinical biomarkers accessible by minimally invasive means is fuelling further biomarker research. Yet, in cancer and other malignancies, translation of candidate markers into sensitive and robust assays is still limited. Exosomes could offer a new route to biomarker discovery, validation and application due to their cell origin-specific cargo and accessibility by minimally invasive sampling (Worst et al., 2017). Zhang et al. describe exosomes as “small particles, big players” as they are also good candidates for generating improved cancer therapies (Zhang et al., 2015).

Exosomes are involved in the complete **cancer** life cycle, including the initiation, growth, progression and drug resistance of tumors (Kosaka et al., 2012; Zhang et al., 2015). Compared to exosomes secreted from healthy cells, a larger amount of exosomes is released from cancer cells, which promotes transformation of local healthy epithelial cells into cancerous cells, subsequently invading the extracellular matrix and contributing to distal metastasis (Tickner et al., 2014). Hoshino et al. reported how tumor-derived exosomes create a favorable microenvironment at future metastatic sites and mediate non-random patterns of metastasis, because the protein content of the tumor determines to a large extent the organotropism (Hoshino et al., 2015).

During the process of epithelial-to-mesenchymal transition (EMT) a cells loses polarity (direction) and cell-cell contacts, thus becoming more motile and gaining invasive properties. These properties prompted researchers to identify a cancerous cell based on stiffness measurements. TGFβ is known to induce the EMT to promote both tumor cell invasion and cell apoptosis. Qin et al. demonstrated how exosomes derived from human breast milk promoted EMT through upregulation of TGFβ2 (Qin et al., 2016). This was shown in both benign and malignant breast cancer cell lines.

Exosomes are also influential in other diseases, such as **neurodegeneration**: Vella et al. demonstrated the propagation of disease-derived exosomes to healthy cells, thereby infecting both neighboring and distant cell types (Vella et al., 2016). More recently, they described involvement of extracellular vesicles in metal homeostasis and neurodenegeration (Bellingham et al., 2015). Nature Biotechnology released a publication in March 2016 celebrating their 20th anniversary by looking at the “greatest hits” (Azvolinsky et al., 2016). Exosomes were ranked twice in the top eight: first, exosomes derived from diseased cells were detected in liquid biopsies using a lab-on-a-chip (Im et al., 2014); a second highlight described exosomes as nanomedicines to deliver drugs across the **blood-brain-barrier** (Alvarez-Erviti et al., 2011). Yet, there are downsides to creating nanomedicines based on exosomes: they are collected after growing mammalian cells to confluency and going through a process of removing cell debris, proteins and microvesicles; although such isolation and purification can be straightforward (see “Exosome Biophysics”), the recovered quantities of exosomes are low and, therefore, the exosomes remain expensive to isolate and culture. This limits the application of exosomes to nanomedicines because of high quantities required for the *in vivo* testing.

### Exosomes in maternal health: breast milk

**Perinatal nutrition** has both immediate and long-term health effects on newborns, babies, infants, children—and likely also—adolescents and adults. This context of prevention, nutrition, and early-life exposures suggests that we should be able to inform women on how to optimize nutrition during pregnancy, in order to: (i) exert positive effects on fetal development and birth outcomes at the level of immune and metabolic health, with possible longer-term effects in infancy, childhood, and adolescence; and (ii) favorably influence breast milk composition and lactation. Maternal nutrition throughout pregnancy and lactation and its impact on maternal health, breast milk quality, and infant health, *via* health-promoting breastfeeding, is a prime case for establishing healthy trajectories early in life.

Exosomes have mainly been researched for their potential as a biomarker source and a delivery system for bioactives. The main application areas are disease diagnostics and drug delivery (see “Exosomes in Health and Disease”). In this section, we bring the **nutrition-and-health** dimension to the exosome context: maternal milk has co-evolved with human and is the gold standard for baby and infant nutrition up to 6–12 months. Proteomic studies of human milk and sub-fractions thereof, such as casein, whey, or the milk fat globule membrane (MFGM) have revealed a plethora of bioactive proteins and peptides beneficial for the developing immune and metabolic system (Casado et al., 2009; Kussmann et al., 2010). By contrast, human milk exosomes are still a largely uncharted proteomic terrain, although we know that exosomes carry cell origin-specific cargo and transport both bioactivity and information between cells. We therefore argue that applying proteomics to (i) human breast milk and its relevance for maternal and infant health, and (ii) the exosome-specific bioactive and cell information content, can synergistically help understand how exosome proteins contribute to maternal and infant immune and metabolic health.

The roles of **exosomes in breast milk** include the regulation of immune response and inflammation. More recently, it was found that they promote epithelial growth in the intestine (Hock et al., 2017). Breast milk significantly decreases the incidence of necrotising enterocolitis in infants. Yet, Hock et al. found rat milk exosomes to enhance epithelial cell proliferation and viability. Liao et al. describe how exosomes can survive harsh conditions, such as digestion, subsequent to which they are taken up by human intestinal cells (Liao et al., 2017). Exosomes provide a stable transportation mechanism for the transfer of miRNA under severe conditions. One study explored exosomes in fermented milk, but, unfortunately, the role of extracellular vesicles released from bacteria was not considered. It remained unclear how this may have influenced the results, because the isolation of bacteria-derived vesicles replicated exosome isolation (Zhang et al., 2015).

The female and male reproductive tracts produce extracellular vesicles that are possibly associated with fertility or infertility and released into urogenital body fluids and mucosae. During pregnancy, placenta-derived EVs can be detected in peripheral blood with changing profiles depending upon the either healthy or pathological progress of the pregnancy. Therefore, these EVs offer a non-invasive diagnostic window into **placental and fetal health**. With this context in mind, Foster et al. reported on extracellular vesicles in blood, milk, and in body fluids of the female and male urogenital tract with an interest in reproductive function (Foster et al., 2016). They summarized the current knowledge about EVs in blood and cord blood, compartments of the male and female reproductive tract, in trophoblast cells from normal and pre-eclamptic pregnancies, in placental *ex vivo* perfusate, in the amniotic fluid, and in breast milk. The syncytiotrophoblast is a major source distributing EVs of fetal origin through the maternal organism and the exosomal composition changes during pregnancy. Exosomes contribute to the maternal body's adaptation to pregnancy. In non-pregnant women, as well as in men, exosomes may improve infertility diagnosis and open novel therapeutic options.

Exosomes have also been investigated with regard to their relevance for **immunological conditions**. For example, differences in human breast milk exosome populations were characterized in relation to allergic sensitization and lifestyle (Torregrosa Paredes et al., 2014). Breast milk was collected from mothers at day 3–8 and at 2 months postpartum. A higher content of exosomes was found in early milk compared to mature milk. Differences in exosome populations were associated with the mother's lifestyle: mothers with an anthroposophic lifestyle produce exosomes that were linked to a lower prevalence of allergic sensitisation. The phenotype of exosomes in breast milk varies with maternal sensitization and lifestyle, which might influence allergy development in the child.

Breast milk exosomes provide a safe passage of miRNA from mother to baby and enhance intestinal epithelial growth in the infant. Both the mammary gland and epithelial cells are controlled by lactogenic hormones, such as prolactin. Major components of the milk fat globule membrane (MFGM) are under the control of these lactogenic hormones, too. Milk fat globule EGF factor 8 (MFG-E8), a major component of MFGM, is upregulated during lactation. MFG-E8 is further upregulated in the involuting mammary gland. MFG-E8 on exosome-like membrane vesicles in milk recovered from post-weaning—so no longer lactating—mammary glands exhibits higher binding activity to phosphatidylserine and apoptotic mammary epithelial cells, and serves as a link between apoptotic mammary epithelial cells and phagocytes (Nakatani et al., 2006). Tolerising exosomes, e.g., tolerosomes, derived from breast milk, were suggested to block an allergic response or prevent allergy development (Admyre et al., 2008).

Melnik et al. wondered whether milk transfers exosomal microRNA promoting thymic regulatory T cell maturation, thereby preventing the development of atopy (Melnik et al., 2014). They suggested milk-derived microRNAs to promote long-term lineage commitment of Tregs downregulating IL-4/Th2-mediated atopic sensitization and effector immune responses. Among exosomal microRNAs, milk also transfers miRNA-155, which is important for the development of the immune system. Infant formulae are deficient in bioactive exosomal miRNAs, in comparison to human breast or cow's milk. The addition of physiological amounts of miRNA-155-enriched exosomes to infant formula for mothers incapable of breastfeeding may offer a new option for preventing atopic disease in infants.

### Exosome biochemistry: biomolecular profiling

Characterization of exosomal cargo is of interest because this molecular content can inform on biogenesis, targeting, and cellular effects of exosomes and may be a source of biomarkers for disease diagnosis, prognosis and response to treatment (Schey et al., 2015). The cargo of exosomes is not a result of a random process, rather it involves a complex sorting mechanism that favors specific biomolecules over others (Abels and Breakefield, 2016; Stevanato et al., 2016). The contents of exosomes have been shown to change when transitioning from health to disease, including conditions like viral infections, neurodegeneration (Alzheimer's, Huntington's), and cancer. The majority of the literature on biomolecular profiling of exosomes reports on RNA and proteins. According to Exocarta (Version 4; http://www.exocarta.org), the largest exosome content database, 4,563 proteins, 194 lipids, 1,639 mRNAs and 764 miRNAs have been identified in exosomes from multiple organisms (Bruschi et al., 2015).

Nucleic acids were first described in exosomes released by mast cells (Valadi et al., 2007). While the mRNAs or miRNAs secreted within exosomes are not random, the exact export mechanism has not yet been experimentally confirmed (Batagov et al., 2011; Colombo et al., 2014). There is growing interest in using miRNAs as biomarkers for disease diagnosis. Encapsulation of miRNAs in exosomes and exosome-like particles confers protection and provides a pathway for intestinal and vascular endothelial transport by endocytosis, as well as delivery to peripheral tissues (Cui et al., 2017). Both the physiological and pathogenic functions of miRNAs, and the fact that they are secreted extracellularly into biological fluids, present miRNAs as promising biomarkers (Cheng et al., 2014).

### Proteomics of exosomes

#### Exosome proteomics technology

Direct comparative analysis of LC-MS/MS runs minimizes sample handling, avoids any chemical protein or peptide modification, and thereby minimizes sample loss due to processing. Therefore, such “label-free” techniques appear to be a suitable strategy for exosome proteomics because: (i) exosomes are precious biophysical preparations with limited total protein amount available; and (ii) the exosome proteome is expected to be vesicle-specific and of lower complexity than e.g., an entire body fluid proteome; this justifies direct peptide/protein quantification entirely based on high LC and MS peptide separation power.

Recent proteomic studies of exosomes have therefore applied quantitative, non-labeled, tag-free workflows (Duijvesz et al., 2013; Hoshino et al., 2015; Wojtuszkiewicz et al., 2016). Spectral counting is a commonly used approach for measuring protein abundance in label-free proteomic analyses (Greening et al., 2017). It accumulates the number of spectra identified for a given peptide in different biological samples and then integrates the results for all measured peptides of the “parent” protein(s) that are to be quantified across those samples (Asara et al., 2008). Specific protocols can be used to focus on spectral counting quantification (Arike and Peil, 2014), with relevance to extracellular vesicles (Tauro et al., 2012, 2013; Amorim et al., 2014). Further details on quantitative mass spectrometry related to comparative analyses can be found in (Griffin et al., 2010).

#### Proteomic surveys of exosomes

Mass spectrometry-based proteomics combined with improved purification schemes for exosomes can immensely contribute to our understanding of the molecular composition and biochemical functions of these extracellular vesicles (Raimondo et al., 2011; Aebersold and Mann, 2016). Exosomes are particularly attractive for proteomic research for the following reasons (Greening et al., 2017).

1. Compared to whole body fluids, like blood plasma or breast milk (Casado et al., 2009; Kussmann et al., 2010), exosome structures are enriched in membrane proteins and low-abundance proteins, which are typically underrepresented in proteomic studies due to their biophysical properties and/or low concentrations.
2. There is a subset of proteins common to all exosomes that is essential for vesicular biogenesis, structure and traffic; this protein set can reveal insights into exosome origin.
3. The presence of specific proteins, related to the study and samples, allows to recognize specific cell types in the greater sample population and, therefore, to identify biomarkers and bioactives. In addition, the analysis of abundance variation of such cell-specific exosomal proteins can shed light onto changes in cellular behavior.

Proteomic cataloging of exosomes from diverse cell types has revealed a common array of membrane and cytosolic proteins, suggesting the evolutionary importance of these membrane particles (Simpson et al., 2009). In addition to this shared group of membrane and cytosolic proteins, exosomes contain distinct subsets of proteins that may be linked to cell-type associated functions (Simpson et al., 2008). Exosomes are rich in endosomal proteins but lack mitochondrial, nuclear, endoplasmic reticulum or Golgi-derived proteins. They have been shown to be enriched with membrane transport and fusion proteins as well as cytoskeletal proteins, such as actin and tubulin. This is expected because exosomes are formed in the cytoplasm. Other exosome proteins include heat-shock proteins, integrins (adhesion proteins) and tetraspanins, such as CD63 (a common exosome marker) (Théry et al., 2002; Vlassov et al., 2012). Eighty % of proteins were found conserved between mouse- and human-derived exosomes (Théry et al., 2002).

#### Proteomics of human body fluid exosomes

There is a considerable body of literature on human body fluid-derived exosomes. Despite our focus on human breast milk as a key such body fluid, but driven by our interest in minimally invasive sampling of exosomes for human (nutritional) studies, we hereunder cite a few seminal papers on exosome proteomics sampling of human blood, urine, and cerebrospinal fluid:

Schey et al. have recently reviewed exosome proteomics with protocols for global qualitative, global quantitative, and targeted quantitative analysis of exosomal proteins. The authors provide a comprehensive tabular summary of proteomic studies of (human) body fluid-derived exosomes, including exosome isolation procedures, exosome marker validations, proteomic techniques, and proteomic results. The cited studies encompass sampling of blood, breast milk, urine, saliva and semen. The article also reports on global quantitative analysis followed by targeted validation of urinary exosome samples taken from human patients, as a clinical example (Schey et al., 2015).

Kalra and coworkers compared different plasma exosome isolation techniques in terms of their suitability for proteomics and assessed the stability of exosomes in human blood plasma (Kalra et al., 2013). They evaluated three exosome isolation techniques: (i) differential centrifugation coupled with ultracentrifugation; (ii) immuno-affinity pull-down with an epithelial cell adhesion molecule; and (iii) OptiPrep™ density gradient separation; with all techniques applied to normal human plasma. Based on quality assessment with mass spectrometry, Western blotting and microscopy, the OptiPrep™ density gradient method isolated exosomal populations in the highest purity, without any detectable contamination of highly abundant plasma proteins. Time-course Western blotting with the exosomal marker TSG101 revealed exosomes to be stable for 90 days in human blood.

Human urinary exosomes have recently been investigated with regard to their protein content exerting metabolic effects (Bruschi et al., 2015). Urinary exosomes can be prepared in large quantities from big volumes of urine, even from a single individual. The whole-urine proteome, the urinary exosome proteome, and the compiled non-urinary exosome proteome (from ExoCharta) were compared. All three proteomes show an enrichment in metabolic pathways related to oxygen utilization and aerobic glucose metabolism. Cytoscape analysis revealed processes involved in aerobic ATP production to be enriched, and therefore likely functional, in exosomes. The authors foresee kidney disease research to benefit from urinary exosome proteomics rather than from global urine proteomics, and expect urinary exosome preparations to mature into a non-invasive clinical sampling alternative to kidney biopsies.

Responding to the suggested relevance of exosome cargo for Alzheimer's disease, Street and collaborators asked first whether human CSF contains exosomes, and, second, whether exosomal protein content varies across individuals (Street et al., 2012). CSF was collected from five donors. The two exosomal marker proteins flotillin 1 and tumor susceptibility gene 101 (TSG101) were identified in the ultracentrifugation pellet using western blot. Mass spectrometric proteomics of these five pellets was performed and revealed large inter-individual variability in terms of both protein amount and composition.

#### Proteomics of human breast milk exosomes

The analysis of human breast milk-derived exosomes by proteomics is still in its infancy. Hereafter, we review a few pioneering studies. Yang et al. deployed iTRAQ proteomics to analyse milk-derived exosomes in **human and bovine colostrum and mature milk** samples (Yang et al., 2017). More than 900 milk exosome proteins were identified and quantified. 575 were differentially expressed between species. The major biological processes covered by these proteins were: response to stimulus, localisation, and cellular component organization. The most prevalent molecular function was “binding.” The identified milk exosome proteins were involved in pathways such as: ribosome; actin cytoskeleton; glycolysis/gluconeogenesis; leukocyte transendothelial migration; aminoacyl-tRNA biosynthesis; pentose phosphate pathway; galactose metabolism; and fatty acid biosynthesis.

Extending beyond proteomic surveys, other authors focused on more specific functions of human milk exosomes: Admyre et al. investigated, whether human breast milk contains exosomes that contribute to **immune regulation** in the infant (Admyre et al., 2007). Based on a label-free LC-MS/MS approach, the authors identified more than 70 proteins present in either colostrum, or mature milk, or both. Larssen et al. published a list of more than 100 proteins detected by means of a proximity extension assay (PEA). The authors used three different protein panels with specificity for **cancer, inflammation** and **cardiovascular** conditions (Larssen et al., 2017).

In the following, we are leveraging these two different, but complementary, approaches (label-free LC-MS/MS vs. PEA) to analyse and understand in more detail the protein composition of human breast milk-derived exosomes. First, we combined two protein lists into one table (Supplementary Table 1), encompassing 188 unique proteins. These proteins were submitted to a KEGG **pathway enrichment analysis** by means of the David Bioinformatics Tools Suite (Huang et al., 2008). The results are summarized in Supplementary Table 2 and show an enrichment of pathways related to the modulation of the immune system, such as “proteoglycans in cancer.” Proteoglycans seem to be involved in the fine-tuning of the innate immune system against pulmonary infections (Wight et al., 2017). In addition, other “response to infection”-related pathways are enriched in human breast milk exosomes, such as “Chagas disease,” “Toxoplasmosis,” or “Influenza A.” These pathway enrichments evidence that human breast milk, and in particular **breast milk exosomes**, provide valuable biological molecules to fight against infections in the early stages of life when the **immune system** is still **maturing**. This finding is also supported by **GO analysis**: Supplementary Table 3 summarizes 13 out of 143 functionally annotated clusters enriched in human breast milk, which are related to the modulation and activation of the immune system.

**Figure 2:**
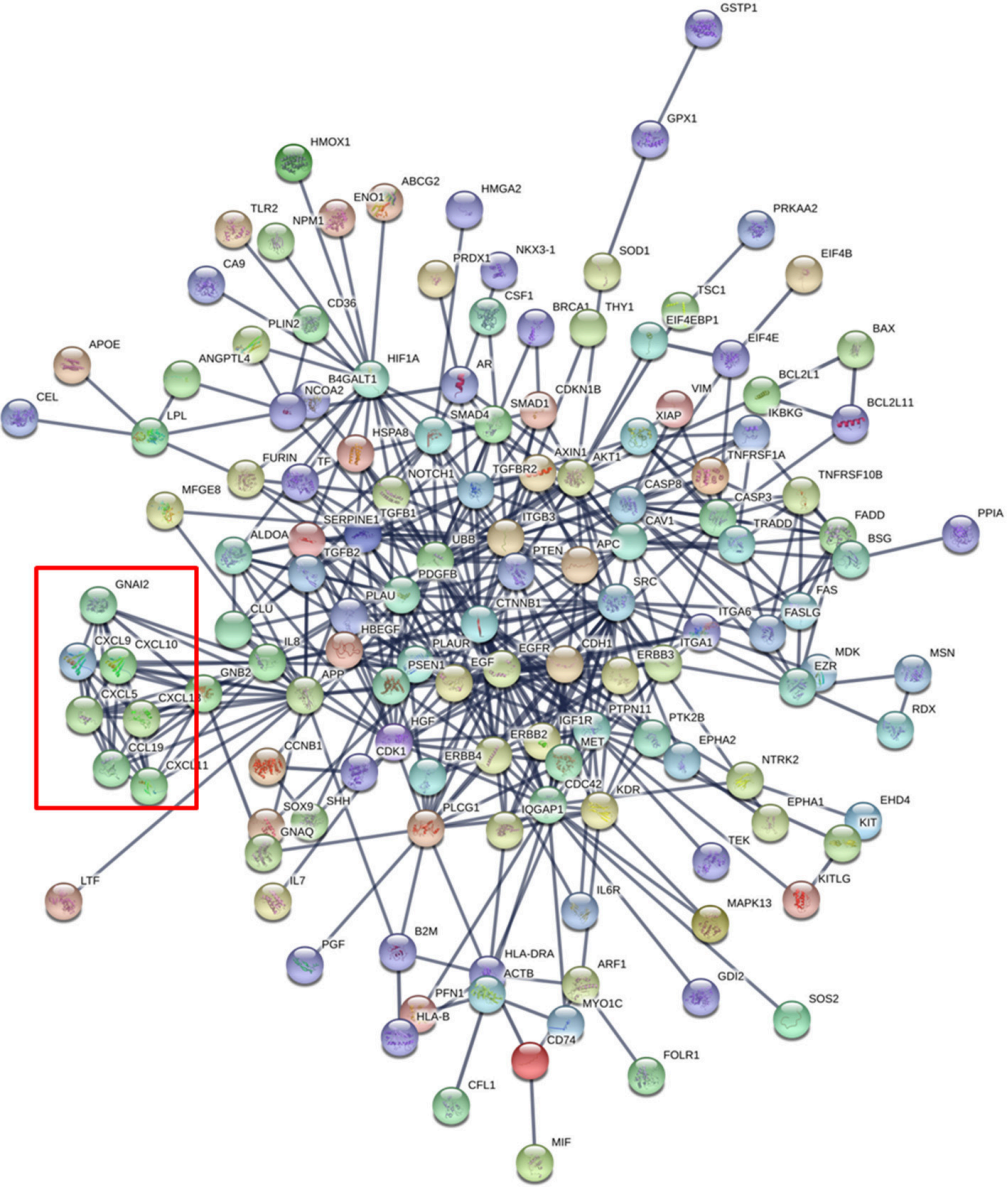
Protein Network generated with the 188 proteins identified in the combined human breast milk exosome proteomics dataset (Admyre et al.,; Larssen et al.). The red square shows a cluster of chemokines.

As an additional dimension to this *in silico* analysis, we show the proteins identified in this combined dataset as tightly interrelated, as depicted in the **protein network** of Figure 2. This network was generated with the online tool STRING-DB (www.string-db.org) considering only the highly confident interactions found in databases that are experimentally confirmed (Szklarczyk et al., 2017). Interestingly, there is a cluster of chemokines showing strong functional links between the protein members (red square in Figure 2) suggesting that these proteins work in a highly coordinated fashion. Globally, the network shows that 28 of the total of 188 proteins show more than 10 interactions (Supplementary Table 4). This cluster of proteins may be the main orchestrator of the **protective effect** in infants against external insults.

The protein composition of milk exosomes provides new information on the potential physiological significance of exosomes to **mammary physiology**. Reinhardt et al. isolated exosomes from the milk of mid-lactation cows and performed LC-MS/MS-based proteomics (Reinhardt et al., 2012). They identified more than 2,000 proteins including all major exosome protein markers. The predominant MFGM proteins (butyrophilin, xanthine oxidase, adipophilin and lactadherin) were found most abundant also in milk exosomes. This said, these proteins accounted for only ca. 1% of the total MS spectra collected from milk exosomes, compared to 15–28% of the total spectra recorded in the MFGM proteome. These data show that the milk exosome secretion pathway differs significantly from that of the MFGM, partly because of the under-representation of MFGM proteins.

#### Micronutrient profling

Whole human breast milk has been extensively studied in terms of micronutrient content and maternal/child health depending on lactation stage, delivery mode, ethnicity, maternal phenotype, baby gender, and social contexts.

- *Lactation stage*: colostrum is high in short-chain human milk oligosaccharides, and low in fat and casein; early milk is high in casein, fat and lactose; and mature milk is again low in fat and also contains less whey protein (Casado et al., 2009).
- *Delivery mode*: vaginal delivery mode was found to be associated with higher protein content in colostrum (Dizdar et al., 2014).
- *Infant gender*: findings are inconsistent as to whether or not infant gender predicts breast milk macronutrient and energy content (Powe et al., 2010; Quinn, 2013).
- *Social environment*: Quian et al. compared macro- and micronutrient composition of breast milk from lactating mothers resident in urban and suburban Shanghai (Qian et al., 2010); Thakkar et al. investigated the dynamics of breast milk composition of Singaporean women with a special focus on lipids (Thakkar et al., 2013).

By contrast, micronutrient profiling of human breast-milk derived exosomes remains a bioanalytically and clinically uncharted terrain (Solomons and Vossenaar, 2013; Koreti and Prasad, 2014). Notably, a PubMed search with the combined set of keywords exosome, breast milk, and (micro)nutrient in both title/abstract and whole text does not yield any entries. The reason for this lack of studies and data is probably two-fold: first, exosomes, including those derived from human breast milk, have to date predominantly been studied at biomolecular level in order to discover protein and RNA biomarkers and to better understand molecular inter-cellular and inter-organism communication. Exosome cargo in terms of nutrients has apparently escaped most of the interest to date. Second, most nutritional research studies have to date focused on single or a few nutrients that may elicit a metabolic effect. These reductionist approaches have resulted in inadequate claims for nutrition as a cure or prevention of disease and disappointment about the real nutritional impact on medicine and health (Monteiro et al., 2015). Rather, multiple factors including environment, host and microbiome, and—most importantly in our context—interactions among nutrients result in nutrient requirements, nutrient bioavailability, and health outcomes. Advanced separation and analytical techniques, such as multi-nutrient profiling (Meisser Redeuil et al., 2015) and metabolomics (da Silva et al., 2016; Guiraud et al., 2017), have only recently enabled the comprehensive and high-throughput analysis of food components and their availability in physiological tissues. The high analytical sensitivity and broad analyte coverage of latest mass spectrometric nutrient and metabolite profiling, combined with a more holistic view of micronutrient status, only now renders exosome micronutrient profiling possible.

In view of exosomes containing biomolecular content specific to cell origin, function and communication, we expect human breast milk exosomes to also contain specific micronutrient compositions, depending on maternal diet and phenotype, as well as physiological interactions between the lactating mother and her breast-fed infant. In fact, numerous studies on maternal micronutrient intake and status/bioavailability (in blood), have been conducted with regard to impact on breast milk micronutrient composition. Yet, they have yielded inconsistent findings, mainly due to incomparability of study design, subject sampling and biomolecular analysis (de Vries, Pundir, de Seymour, McKenzie, Kussmann; submitted). By contrast, studies comparing maternal nutrition with micronutrient content of breast milk exosomes are entirely lacking. We therefore strongly argue to integrate comprehensive biomolecular exosome profiling into today's systems nutrition studies, which are now enabled by comprehensive micronutrient analysis and metabolomics. In doing so, we can enhance and complement insights into maternal nutrient status and breast milk quality, depending on maternal, infant and environmental factors.

## Conclusions

Exosomes are biological, vesicular nanostructures that transport information and bioactivity within and between cells. Their biophysical isolation in purity and integrity is delicate and their biophysical characterisation requires complementary methods, depending on the biofluid and cell type of origin. Exosomes have to date mainly been researched for their potential and value in drug delivery and biomarker discovery. We have placed exosomes into the context of other vesicles and reviewed their biophysical features, which guide their isolation procedures. We have summarized the roles and functions of exosomes in disease conditions, with a focus on cancer and immunology. The main objective of our article is to open the nutritional dimension of exosome research with a particular focus on human breast milk-derived exosomes and their relevance and function for maternal and infant health. Extending from citations and a pure review article, we have combined two proteomic datasets from pioneering human breast milk exosome studies. We thereby showcase the enrichment of pathways involved in early-life immunity as evidenced by the high number and connectivity of immune function-related proteins found in human breast milk exosomes. In view of these promising first proteomic insights, and with micronutrient profiling virtually non-existing, we argue that both proteomics and micronutrient analysis of human breast milk exosomes will add great value to the understanding of exosomes as deliverers of bioactives, highly relevant to breast milk quality, and the health of the mother and infant.

## Author contributions

All authors contributed to the writing of the manuscript. CdlT and MK focused on the proteomics, micronutrient and human breast milk (biochemistry, nutrition, and bioanalytics). RVG and TN focused on introducing exosomes and biophysics. JB focused on proteomics and immunology.

### Conflict of interest statement

The authors declare that the research was conducted in the absence of any commercial or financial relationships that could be construed as a potential conflict of interest.

## Footnotes

^1^Exosome Explosion | The Scientist Magazine® The Scientist. Available online at: http://www.the-scientist.com/?articles.view/articleNo/30793/title/Exosome-Explosion/ [Accessed March 30, 2017].

## Supplementary material

Supplementary Table 1 Combined list of 188 proteins identified in human breast milk-derived exosomes by Admyre et al. (2007) and Larssen et al. (2017).

Supplementary Table 2 Significantly enriched KEGG pathways (*P*-value < 0.001) in human breast milk exosomes according to proteins identified by Admyre et al. (2007) and Larssen et al. (2017).

Supplementary Table 3 The 13 most relevant functional annotation clusters (of a total of 143) found to be enriched in the combined data set of human breast milk exosome proteomes by Admyre et al. (2007) and Larssen et al. (2017).

Supplementary Table 4 Number of interactions found for each protein of the protein network generated from the 188 proteins identified in the combined human breast milk exosome proteomics dataset (Admyre et al., 2007; Larssen et al., 2017).
